# Genetic mutations and prognostic indicators in differentiated thyroid cancer: a molecular perspective

**DOI:** 10.55730/1300-0144.5944

**Published:** 2024-12-25

**Authors:** Halim ÖZÇEVİK, Müge ÖNER TAMAM, Gündüzalp Buğrahan BABACAN, Selma ŞENGİZ ERHAN, Merve Nur ACAR TAYYAR, Biray ERTÜRK

**Affiliations:** 1 Department of Nuclear Medicine, Hamidiye Medical Faculty, University of Health Sciences, İstanbul, Turkiye; 2 Department of Nuclear Medicine, Prof. Dr. Cemil Taşcıoğlu City Hospital, University of Health Sciences, İstanbul, Turkiye; 3 Department of Pathology, Prof. Dr. Cemil Taşcıoğlu City Hospital, University of Health Sciences, İstanbul, Turkiye; 4 Department of Medical Genetics, Prof. Dr. Cemil Taşcıoğlu City Hospital, University of Health Sciences, İstanbul, Turkiye

**Keywords:** Differentiated thyroid cancer, BRAF, RAS, gene mutation, iodine-refractory disease, prognostic biomarkers

## Abstract

**Background/aim:**

The aim of this study was to investigate the relationship between the presence of the BRAF, HRAS, NRAS, and KRAS gene mutations and the development of dedifferentiation (iodine-refractory disease) and extrathyroidal disease in patients with differentiated thyroid carcinoma (DTC).

**Materials and methods:**

The patient group included 77 adults classified as intermediate or high-risk according to the American Thyroid Association’s 2015 guidelines who underwent total thyroidectomy followed by radioiodine I-131 (RAI) therapy between June 2014 and December 2022. Clinical data were collected via the hospital information system, including the number of surgeries and RAI treatments and the levels of thyroglobulin (Tg), anti-thyroglobulin, and thyroid-stimulating hormone. The histopathological subtypes of DTC were reevaluated, and mutation analyses of the BRAF, KRAS, NRAS, and HRAS genes were performed using real-time polymerase chain reaction (PCR). Statistical analyses were conducted using Medcalc software, with p < 0.05 considered significant.

**Results:**

Of the 77 patients, most had classical papillary thyroid carcinoma, while others represented various subtypes. No mutations were found in BRAF K601E/V600_K601, KRAS G12x-G13D, or NRAS G12-G13; however, NRAS Q61x was found in one patient, HRAS Q61x in 12, and BRAFV600E/Ec in 36. A significant relationship was observed between HRAS Q61x mutation and disease response, alongside a significant association between gene mutations and iodine-refractory disease development (p = 0.0004). A ROC curve analysis indicated a 49.2 ng/mL threshold for Tg with 75% sensitivity and 94.1% specificity.

**Conclusion:**

The findings suggest that the HRAS Q61x gene mutation is significantly associated with iodine-resistant disease. It may serve as a prognostic biomarker in early-stage thyroid cancer and aid in disease monitoring in metastatic patients.

## 1. Introduction

Thyroid cancer is the most common endocrine malignancy, with a relatively good prognosis after early diagnosis and treatment [1–5]. Papillary thyroid cancer (PTC) is the most prevalent form of thyroid cancer, accounting for more than 90% of all thyroid cancer diagnoses. While the majority of PTC patients experience favorable outcomes and clinical indolence, a small proportion exhibits aggressive clinical characteristics and a poor prognosis [3–4]. This subset of PTCs may present with lymph nodes and distant metastases, resulting in higher mortality rates and a worse prognosis [3,5]. Therefore, risk stratification is crucial to identify high-risk patients who may experience recurrence and to facilitate more aggressive management and monitoring strategies [1–5].

Practice guidelines recommend a treatment algorithm that includes surgical resection followed by radioactive iodine therapy; conventional chemotherapy and radiation therapy have been shown to be ineffective [6–7]. Molecular testing modalities are also offered clinically to aid surgical and therapeutic decisions for advanced or metastatic thyroid cancers [8–9]. Early identification of molecular markers in primary tumors may help predict radioiodine I-131 (RAI) therapy avidity and the response of metastatic lesions in order to identify patients who may benefit most from other systemic therapies [10]. However, the significant role of molecular mechanisms in distant metastases in differentiated thyroid carcinoma (DTC) has yet to be systematically studied. Recognizing these molecular biomarkers could serve as crucial indicators of tumor behavior and disease prognosis.

Notably, genetic analyses have revealed common mutations associated with thyroid cancer, such as BRAFV600E and RAS-like mutations (HRAS, KRAS, and NRAS). BRAFV600E mutation occurs frequently in PTCs, particularly in advanced cases, potentially contributing to enhanced cancer cell proliferation. Nonetheless, the relationship between BRAFV600E mutations, aggressive clinicopathologic features, and poor outcomes remains variable and controversial [2,3,9].

Studies have shown that the presence of BRAF V600E is associated with more aggressive tumor behavior, such as extrathyroidal extension, lymph node metastasis, and poor prognosis. Although it is an important marker for risk stratification, the clinical impact of this mutation may be influenced by additional genetic or environmental factors, and its predictive value can vary among patients [11–14]. Some studies have associated the cooccurrence of RAS-like mutations with the BRAF V600E mutation with worse outcomes [15–16]. However, few studies have investigated these mutations’ associations with metastatic disease and radioiodine-refractory (RAIR) disease [17–20]. This study was designed with the aim of examining the relationship between clinicopathological characteristics and mutations in the BRAF, HRAS, KRAS, and NRAS genes with metastatic disease and RAIR development in patients with differentiated thyroid cancer.

## 2. Methods

### 2.1. Patient selection

Between 1 June 2014 and 31 December 2022, 78 adult patients who received RAI treatment after total thyroidectomy, whose total thyroidectomy pathology preparations were available, who had no history of secondary malignancy before and after the diagnosis of thyroid cancer, and who were intermediate or high risk according to the risk classification criteria of the American Thyroid Association (ATA) 2015 guidelines were enrolled in this prospective study. Postoperative data on tumor characteristics included tumor size and type, tumor extension, multicentricity, extrathyroidal extension, lymph node metastasis, lymphatic invasion, soft tissue, and vascular invasion. Patients were selected in a 2:1 ratio of those with/without known extrathyroidal disease between the groups with an alpha value of 0.05 and 80% power in the chi-square test as a result of power analysis. The study was completed with 77 patients after one patient withdrew. The follow-up data of the patients were accessed through the hospital information management system, including data on the number of operations performed, the number and doses of RAI administered, thyroglobulin (Tg), anti-thyroglobulin (anti-Tg), and thyroid stimulating hormone (TSH) before treatment and during follow-up periods. With this information, follow-up classification was done following the dynamic disease assessment classification of the ATA 2015 guideline.

### 2.2. Dynamic follow-up classification

The dynamic risk stratification criteria used for follow-up after total thyroidectomy and RAI in differentiated thyroid cancers are as follows:

- excellent response: negative imaging and either suppressed Tg < 0.2 ng/mL or TSH-stimulated Tg < 1 ng/mL,
- biochemically incomplete response: negative imaging and suppressed Tg ≥ 1 ng/mL or stimulated Tg ≥ 10 ng/mL or rising antiTg antibody level,
- structurally incomplete response: structural or functional evidence of disease with any Tg level with or without antiTg antibodies,
- indeterminate response: nonspecific findings on imaging, faint uptake in the thyroid bed on RAI scanning, nonstimulated Tg detectable but <1 ng/mL, and stimulated Tg detectable but <10 ng/mL.

### 2.3. Investigation of pathological findings

To determine the histopathological subtypes of DTC, hematoxylin eosin (HE) stained slides for all tumors of each case were reevaluated. For the molecular study, four sections of 5-μ thickness were taken from the selected paraffin block and placed into an Eppendorf tube. The sections were reexamined and identified according to the World Health Organization (WHO) 2022 diagnostic criteria for endocrine and neuroendocrine tumors in the Medical Pathology Department of Prof. Dr. Cemil Taşcıoğlu City Hospital.

### 2.4. Investigation of gene mutations

After the selection of appropriate tissue samples from the tumors, a real-time polymerase chain reaction (PCR) test was used for mutation analysis of BRAF codons 600 and 601, KRAS codons 12–13 and 61, NRAS codons 12–13 and 61, and HRAS 12–13 and 61 genes. The genetic analyses were performed using the QIAGEN EasyPGX (Diatech Pharmacogenetics., Dublin, Ireland) genetic mutation analysis panel kit. The Delta Cq method was used to analyze the data obtained, and a Delta Cq calculation of the mutation mix and the control mix of the samples was performed.

### 2.5. Determination of radioiodine-refractory disease status

According to the ATA 2015 guidelines, RAIR DTCs are identified by:

- the presence of malignant/metastatic tissue that never concentrates RAI,
- the presence of tumor tissue that loses the ability to concentrate RAI after previous evidence of RAI-avid disease,
- RAI uptake in some lesions but not in others, and
- metastatic disease that progresses despite a significant concentration of RAI.

### 2.6. Statistical analysis

The data were analyzed using MedCalc Statistical Software v.22.016 (MedCalc Software, Ostend, Belgium). A chi-square test was used to analyze the categorical variables and the presence of metastatic disease, and the Kaplan–Meier survival analysis was used to analyze the survival relationship between the development of iodine refractory status and genetic test results. A Cox regression analysis was performed to determine whether any associations exist between the mutations and RAIR disease. An ROC curve graph was used to determine the threshold values of continuous variables. Results with p < 0.05 were considered significant.

## 3. Results

Of the 77 patients included in the study, 49 (63.6%) were female, 28 were male, and the mean age was 46.66 years. The data of continuous variables of the patients are summarized in Table 1. There were 67 papillary carcinomas, seven invasive encapsulated follicular variant papillary carcinomas, two oncocytic carcinomas, and two high-grade follicle cell-derived carcinomas. In the subtype analyses, 54 patients were in the classical subtype of PTC, seven were in the oncocytic subtype PTC, seven were in the invasive encapsulated subtype PTC, four were in the infiltrative follicular subtype PTC, one was in warthin-like PTC, one was in tall cell PTC, and three were in solid/trabecular/insular differentiated PTC subtypes. While two patients were defined as oncocytic carcinoma, their pathology preparations were found to be compatible with poorly differentiated PTC according to the WHO 2022 classification. The pathological data of the patients after total thyroidectomy are summarized in Table 2.

In the genetic panel applied in the study, BRAF K601E/V600_K601, KRAS G12x-G13D, KRAS Q61x, and NRAS G12-G13 gene sequences were not detected in any patient included in the study. An NRASQ61x mutation was detected in one patient, an HRASQ61x mutation in 12 patients, and a BRAFV600E/Ec mutation in 36 patients. In the statistical analysis performed to examine the relationship between post-follow-up evaluation and genetic analysis, 4 of 12 patients with a HRASQ61x mutation had a perfect response at follow-up. In comparison, the structural disease was detected in eight patients. In the patient group without mutations, 36 patients had excellent responses, eight had indefinite responses, eight had incomplete biochemical reactions, and 13 had structural diseases. The BRAFV600E/Ec and HRASQ61x mutation statuses are summarized in Table 3.

According to the follow-up data, 10 patients (13%) had RAIR disease, 19 (24.7%) had metastatic disease during the disease period, 11 had elevated antiTg (14.3%), and six (7.8%) patients died. The median follow-up period was 30 (12–108) months. A chi-square analysis showed a statistically significant difference between the groups (p = 0.0074). A chi-square analysis was performed to analyze the relationship between metastatic disease status and the BRAFV600E/Ec mutation at the end of the median follow-up period of 30 months, and it revealed no statistically significant relationship between this mutation and metastatic disease (p = 0.3216). When the same analysis was performed for the HRASQ61x mutation, there was a statistically significant relationship between the groups (p = 0.0035, χ^2^ = 8.552). While 7 of 12 patients with the HRASQ61x mutation had evidence of metastatic disease during follow-up, 12 of 65 patients without this mutation had evidence of distant metastasis.

Fisher’s exact test, which was used to examine the relationship between histopathological subtypes of tumors and metastatic disease status, revealed a statistically significant relationship between the subtypes and metastatic disease (p = 0.017).

A statistically significant relationship was found from Fisher’s exact test used to examine the relationship between histopathological subtype and genetic mutations (p = 0.046). In this analysis, this mutation was present in both of the two patients diagnosed with poorly differentiated PTC (2/2), in two of the three patients diagnosed with solid trabecular subtype PTC (2/3), in six of the classical subtype PTC patients (6/50), and in one patient each of oncocytic subtype PTC and invasive encapsulated subtype PTC (1/7, 1/7). This genetic mutation was not detected in the other subtypes. When Fisher’s exact test was performed between BRAFV600E/Ec and the histopathological subtypes, it identified a statistically significant association (p < 0.001, χ^2^ = 26.08). There were 27 patients (27/50) in the classical subtype PTC group, seven (7/7) in the oncocytic subtype PTC group, and one patient each in the whartin-like and tall cell subtype groups (1/1, 1/1) with this gene mutation, but this mutation was not detected in patients with other subtypes. While three patients with both mutations were diagnosed as classical subtype PTC, one was diagnosed as oncocytic subtype PTC.

**Figure 1:**
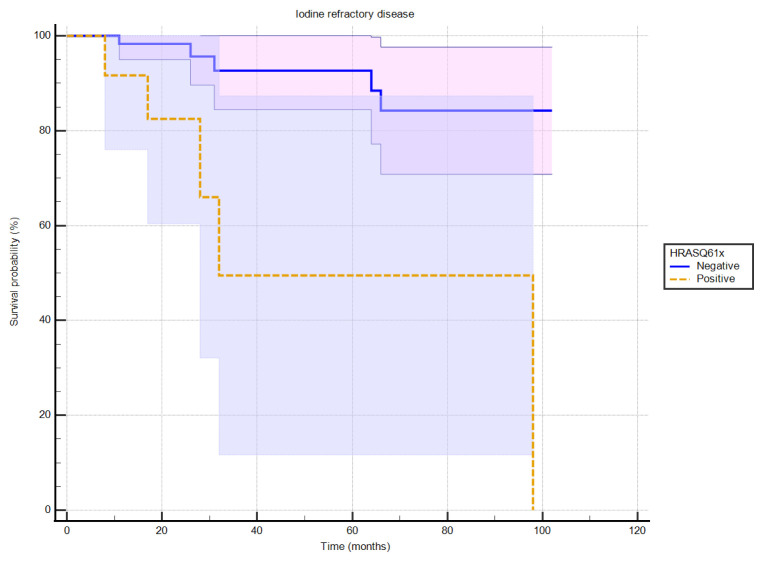
The association between the HRASQ61x gene mutation and iodine refractory survival.

Of the 36 patients with the BRAFV600E/Ec mutation, 18 had an excellent response, six had an indeterminate response, two had an incomplete biochemical response, and 10 had structural disease. In the patient group without this mutation, 22 patients had an excellent response, two had an indeterminate response, six had incomplete biochemical responses, and 11 had structural disease. In the group without this mutation, 40 patients had an excellent response, eight had an indeterminate response, eight had an incomplete biochemical response, and 10 had structural disease findings. The chi-square test revealed no statistically significant relationship between the treatment response groups regarding the presence or absence of the BRAFV600E/Ec mutation (p = 0.247). In addition, Kaplan–Meier survival analysis performed for the analysis of RAIR disease and gene mutations showed a statistically significant relationship between these mutations and the development of RAIR disease (p = 0.0008) (Figure 1). RAIR disease was observed in 5 of 12 patients with the HRASQ61X mutation, and it developed in 5 of 65 patients without this mutation. When the same analysis was performed for the BRAFV600E/Ec mutation, no statistically significant correlation was found between this mutation and the development of RAIR disease. Three patients with the BRAFV600E/Ec mutation developed iodine refractory status, while 33 patients did not. RAIR disease developed in 7 of 41 patients who did not have this mutation (p = 0.4421). A statistically significant relationship was found from the Cox regression analysis performed to examine the effect of the coexistence of both mutations on the development of iodine refractory status (p = 0.0021, Harrell’s C-index = 0.703, HR: 3.85, 95% CI of Exp(b) 1.0029 to 32.9179). In this analysis, no mutation was detected in the genetic analysis performed in 4 of 10 patients who developed RAIR disease, while the presence of HRASQ61X in three patients, BRAFV600E/Ec in one patient, and both of these mutations together in two patients was detected.

Among the ten patients who developed iodine refractory status, three had the solid trabecular subtype, two had the HRASQ61X mutation, and no mutation was found in the other patient. Five patients were classified in the classical subtype, two of whom were positive for both mutations, one was positive only for BRAF V600E mutation, and two had no mutation. One of the other two patients with iodine refractory status was diagnosed with invasive encapsulated follicular variant PTC, and no mutation was detected. In contrast, the last patient was diagnosed with poorly differentiated thyroid cancer, and only the HRASQ61X mutation was positive in this patient.

In the univariate Cox regression analysis performed to examine the development of RAIR disease and all other variables obtained from the pathology data, none of the variables of extrathyroidal extension, lymphovascular invasion, lymph node metastasis, or thyroid capsule invasion were statistically significant (p = 0.695, p = 0.648, p = 0.964, and p = 0.240, respectively).

**Figure 2:**
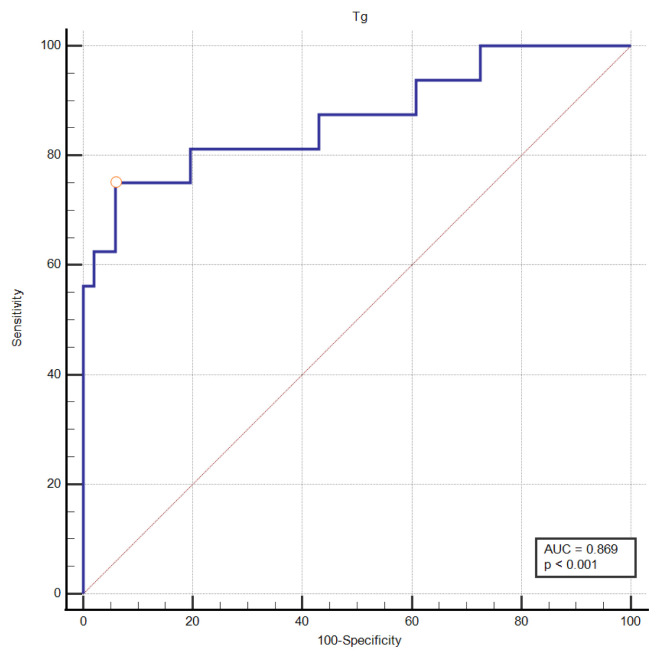
The ROC curve for serum-stimulated Tg value metastatic disease status.

In addition, in the ROC curve analysis performed to examine the relationship between the presence of metastatic disease and Tg, the threshold value was determined as 49.2 ng/mL with 75% sensitivity and 94.1% specificity (AUC: 86.9%, p < 0.001) (Figure 2).

## 4. Discussion

In this study, the genetic and clinical characteristics of 77 patients with thyroid cancer were analyzed, focusing on histopathological subtypes, genetic mutations, and clinical outcomes. Specifically, the relationships between the development of dedifferentiation, manifested as iodine-refractory disease, and mutations in the BRAF, HRAS, NRAS, and KRAS genes were investigated. The findings revealed a significant association between the HRASQ61X mutation, the development of iodine-refractory disease, and the persistence of structural disease following standard treatments. These results suggest that identification of the HRASQ61X mutation in early-stage thyroid cancer may be a valuable prognostic biomarker for predicting treatment resistance and disease progression.

The study also highlighted the role of histopathological subtypes in thyroid cancer prognosis. Solid/trabecular subtypes and poorly differentiated carcinomas were strongly associated with metastatic disease, reinforcing the importance of tumor subtypes in predicting disease progression (p = 0.017). Furthermore, a significant relationship was found between specific mutations and histopathological subtypes, particularly the HRASQ61X mutation in poorly differentiated and solid trabecular PTC subtypes. These findings suggest that the molecular profile of a tumor, along with its histopathological features, could provide valuable insights into its clinical behavior and guide more personalized treatment strategies [12].

This study also found that Tg is a reliable, noninvasive marker for early detection of metastasis in differentiated thyroid carcinoma, consistent with previous studies [3,4]. Elevated Tg levels indicate recurrent or metastatic disease and are vital for postoperative management and long-term follow-up [3,4]. Regular Tg monitoring helps identify at-risk patients, though Tg may not reflect disease burden in all cases [15]. Recently, the BRAF V600E mutation has become a commonly used biomarker for thyroid cancer recurrence. Despite its wide use, BRAF V600E alone may not consistently predict biochemical behavior or disease burden in all patients. While studies, including Liu et al., have linked BRAF V600E to worse outcomes and increased recurrence [15], Nikiforov et al. suggested that the clinical impact of this mutation may be modulated by other genetic and environmental factors [12]. This underscores the need for a multifactorial approach to assessing DTC prognosis and recurrence risk.

This study found no statistically significant association between the BRAF V600E/Ec mutation and any of the five clinical markers (largest foci diameter, age, Tg, antiTg, and Tg/TSH), suggesting that this mutation does not strongly correlate with key clinical features like tumor size or biomarker levels (p > 0.05). These findings diverge from several studies that have linked BRAF V600E to more aggressive thyroid cancer characteristics, such as larger tumors and higher recurrence rates [9,11]. However, the absence of a significant relationship in this study could be explained by the variability of BRAF V600E’s impact across different subtypes of PTC and the potential influence of coexisting molecular alterations [13].

The BRAFV600E/Ec mutation was detected in 36 patients, a significant proportion of our study population, particularly in the classical and oncocytic subtypes of PTC. While this mutation has been generally associated with worse outcomes and higher recurrence rates in previous studies [13,14], this study, unlike for HRASQ61X, did not reveal a statistically significant association between BRAFV600E/Ec and metastatic disease (p = 0.3216) or iodine-resistant status (p = 0.4421). Some metaanalyses have also reported no association between the BRAF V600E mutation and distant metastasis, disease persistence, or recurrence [25–27]. Lai et al. reported that although patients with mutated BRAF V600E have higher-risk histological subtypes, this mutation does not predict long-term outcomes in PTC, which is similar to this study. They found no association between BRAF mutations and distant metastasis. They also emphasized that further genetic analysis is needed for BRAF-negative patients to identify potential molecular markers associated with poor PTC prognosis, including RAS mutations, RET fusions, NTRK fusions, and TERT promoter mutations [28].

These results challenge previous studies demonstrating a link between BRAFV600E and aggressive disease features such as extrathyroidal extension and recurrence [11]. However, variability in the impact of this mutation might stem from differing tumor subtypes or coexisting mutations, which could influence its clinical significance [13]. Despite the lack of strong associations in this cohort, BRAFV600E is still regarded as a key mutation in thyroid cancer.

The metaanalysis by Ellisie et al. showed that BRAF mutation status was associated with the recurrence of papillary microcarcinomas, highlighting the potential benefit of genotyping in preoperative and postoperative planning. Identification of a BRAF mutation may aid in the risk stratification of patients with papillary thyroid metastatic cancer for surgical management and observation [14].

An important aspect of this study is the responses to treatment of patients with BRAFV600E/Ec. Although 18 patients had excellent responses, 10 developed structural diseases, suggesting that BRAFV600E is not universally predictive of poor outcomes. These results align with prior studies indicating that BRAFV600E is a significant factor in predicting disease recurrence. However, its impact can vary depending on other factors, such as histopathological subtypes or coexisting genetic alterations [7,14].

This study suggests that the HRASQ61 mutation is significantly associated with the greatest tumor diameter (p = 0.022). The median tumor diameter in patients with the HRASQ61 mutation was notably larger than 16 mm. This finding implies that larger tumors are more likely to harbor the HRASQ61 mutation, suggesting that tumor size may indicate this genetic alteration. The relationship between HRASQ61 and tumor size also holds clinical relevance. Larger tumors are typically associated with more advanced disease, increased risk of local invasion, and higher likelihood of metastasis [21]. Identifying HRASQ61 mutations in patients with larger tumors could help clinicians anticipate aggressive disease behavior and guide targeted therapeutic interventions. For example, HRAS-mutated tumors may not respond as effectively to conventional therapies like RAI, underscoring the need for alternative treatment approaches, such as kinase inhibitors, for these patients [19].

A significant finding of this study is the strong association between the HRASQ61X mutation and metastatic disease. Seven of 12 patients with this mutation developed metastases, compared to 12 of 65 patients without the mutation in our study. The chi-square analysis (p = 0.0035) demonstrated a statistically significant relationship between HRASQ61X and metastasis. This corroborates previous research indicating that RAS mutations, especially those involving codon 61, are linked to more aggressive thyroid cancer phenotypes [11,17]. The presence of this mutation in poorly differentiated and solid trabecular subtypes further reinforces its role as a marker of aggressive tumor behavior, echoing similar findings in recent studies that highlight HRASQ61X as a critical prognostic indicator for poor outcomes [19,21]. HRASQ61X may be a valuable marker for identifying patients requiring more intensive monitoring and aggressive treatment strategies. The significant association between HRASQ61X and metastatic disease in this study also aligns with findings from Schlumberger et al., who highlighted the potential for targeted therapies in RAS-driven cancers [22]. Given that HRASQ61X mutations are linked to a higher metastatic potential, using novel targeted therapies, such as kinase inhibitors, might offer improved outcomes for patients with this genetic profile [23]. The 2022 WHO classification also highlights that RAS-driven tumors, including those with HRASQ61X, tend to behave more aggressively and may require a distinct approach from BRAF-mutated thyroid cancers [24]. As thyroid cancers with the HRASQ61X mutation tend to be more aggressive, these patients may benefit from more personalized and intensified treatment strategies.

A notable focus of this study was the development of RAIR disease, which occurred in 13% of the patients. The HRASQ61X mutation was significantly associated with this condition (p = 0.0008), further solidifying its importance as a prognostic marker. Specifically, 5 out of 12 patients with HRASQ61X developed iodine-refractory disease, compared to only 5 out of 65 patients without the mutation. This finding is consistent with previous reports, which have also linked RAS mutations, particularly in codon 61, to resistance to radioactive iodine therapy [19,21]. The Kaplan–Meier analysis further confirmed the role of HRASQ61X in developing iodine-resistant disease, suggesting that patients with this mutation should be closely monitored for potential resistance to RAI therapy.

Liu et al. supported these findings by noting that while most PTC patients can be cured through surgery and RAI ablation, a small subset progresses to RAIR thyroid cancer. Early prediction of RAIR is crucial for improving patient outcomes, and Liu’s study highlighted the potential of blood biomarkers in predicting RAIR thyroid cancer [15]. These insights align with this study’s data, reinforcing the importance of identifying early molecular markers such as HRASQ61X for targeted monitoring and intervention.

Interestingly, no significant relationship was found between the BRAFV600E mutation and iodine-refractory disease in our cohort. Although three patients with BRAFV600E/Ec developed iodine-refractory disease, most did not, suggesting that this mutation alone may not be a reliable predictor of iodine resistance. This finding contrasts with previous studies, such as that by Xing et al., which reported an association between BRAFV600E and an increased likelihood of iodine-refractory disease [11]. However, Simões-Pereira et al. emphasized that early identification of molecular markers can help predict RAI therapy responses and identify patients who may benefit from alternative systemic therapies, highlighting the complexity of the mutation’s clinical impact [29].

This study has several limitations. Clinical characteristics were collected from notes and reports without comprehensive reevaluation of all imaging. Consecutive patients were not eligible to participate in the study since it only included those who had had a full thyroidectomy and/or lymph node dissection and whose pathology samples were accessible. Furthermore, the pathology results were reevaluated by a single pathologist. This prospective study examined BRAF codons 600 and 601, KRAS codons 12–13 and 61, NRAS codons 12–13 and 61, and HRAS 12–13 and 61 genes, but fusion genes such as the TERT promoter mutation were not included due to cost. Comprehensive studies must be conducted with a broader panel of gene mutations covering the entirety of thyroid cancer.

## 5. Conclusion

This study highlights the prognostic significance of the HRASQ61X mutation in thyroid cancer, mainly its association with metastasis and iodine-refractory disease. Furthermore, although the BRAFV600E/Ec mutation does not consistently correlate with most clinical markers, its association with extrathyroidal extension and specific pathological subtypes suggests its potential role in disease progression. However, it was not statistically significant in this cohort, particularly in predicting iodine-refractory status. These findings provide valuable insights for personalized treatment strategies and risk stratification in thyroid cancer patients. Further research is warranted to fully understand the molecular mechanisms underlying these mutations and their influence on treatment outcomes.

## Figures and Tables

**Table 1:** Patient characteristic data.

Variables	Maximum	Minimum	Median	Mean	SD
**Age**	77	18	46.5	46.66	15.66
**Tumor size (mm)**	90	2	18	24.46	19.35
**Stimulated TSH**	198	6.58	64.26	70.09	37.83
**Stimulated Tg**	15998	0.04	5.89	325.57	1969.24
**Dose (total)**	850	50	150	201.95	134.61
**Tg (follow-up)**	1774	0.04	0.09	39.95	217.08
**TSH (follow-up)**	11.7	0.01	0.36	0.91	1.83

SD = standard deviation, TSH = thyroid stimulating hormone, Tg = thyroglobulin.

**Table 2:** Pathological results of surgery.

Variables	Pathologic findings	n	%
Multicentricity	−	28	36.36
	+	49	63.64
Lymph node metastasis	−	35	45.45
	+	42	54.55
Lymphovascular invasion	−	18	23.38
	+	59	79.22
Extrathyroidal extension	−	47	61.04
	+	30	38.96

**Table 3:** Gene mutation status of patients.

Mutation	Status	n	%
BRAFV600E/Ec	+	36	46.8
	−	41	53.2
HRASQ61x	+	12	15.6
	−	65	84.4
Overall	Negative	34	44.2
	HRASQ61	7	9.1
	BRAFv600E/Ec	31	40.3
	Comutation	5	6.5

## Data Availability

The data used in the study are available on request from the corresponding author.
